# Hydroxyacid Oxidase 2 (HAO2) Inhibits the Tumorigenicity of Hepatocellular Carcinoma and Is Negatively Regulated by miR-615-5p

**DOI:** 10.1155/2022/5003930

**Published:** 2022-04-28

**Authors:** Yuxuan Li, Mingchao Zhang, Xiuling Li, Yanyan Wang, Yu Wang, Yuan Li, Huihui Zhu, Hui Ding, Xiaofang Li, Suofeng Sun

**Affiliations:** ^1^Department of Gastroenterology, Zhengzhou University People's Hospital, Henan Provincial People's Hospital, Zhengzhou, Henan 450003, China; ^2^Xinxiang Medical University, Xinxiang, 453000 Henan, China; ^3^Department of Traditional Chinese Medicine, The Third Affiliated Hospital Affiliated of Henan University of Traditional Chinese Medicine, Zhengzhou, Henan 450003, China; ^4^Department of Gastroenterology, School of Clinical Medicine, Henan University, Henan Provincial People's Hospital, Zhengzhou, Henan 450003, China; ^5^Zhengzhou University People's Hospital, Henan Provincial People's Hospital, Zhengzhou, Henan 450003, China

## Abstract

**Background:**

Hepatocellular carcinoma (HCC) is the sixth most common kind of cancer worldwide and the third leading cause of cancer mortality. Although a few studies have shown that hydroxyacid oxidase 2 (HAO2) may prevent HCC development, the molecular mechanism is unclear.

**Methods:**

We examined the levels of HAO2 expression in 23 pairs of HCC/paracancerous tissues by quantitative real-time polymerase chain reaction (qRT-PCR) and evaluated HAO2's expression in The Cancer Genome Atlas (TCGA) database. Furthermore, we examined the biological activity of HAO2 utilizing cell-based functional assays. Additionally, we evaluated the relationship between miR-615-5p and HAO2 in Hep3B cells using a dual-luciferase reporter system and assessed the downstream regulatory mechanisms of miR-615-5p on HAO2. Finally, the nude mice tumor formation experiment was used to determine the impact of HAO2 on the tumorigenicity of HCC cells.

**Results:**

HAO2 expression was considerably underexpression in HCC tissues and cells, and patients with low HAO2 expression had poorer disease-free survival. Inhibition of cell proliferation, migration, and invasion was observed when HAO2 was overexpressed. miR-615-5p had a negative relation with HAO2, and miR-615-5p restored HAO2's biological activity in HCC cells. Additionally, the tumor volume and weight were considerably reduced in the OV-HAO2 group compared to the OV-NC group.

**Conclusion:**

HAO2 was found to be underexpressed in HCC tissues and cells, and HAO2 overexpression inhibited HCC cell motility, which was negatively regulated by miR-615-5p. Exogenous expression of HAO2 reduced the tumorigenicity of HCC cells in vivo in nude mice.

## 1. Introduction

Hepatocellular carcinoma (HCC) is the sixth most often diagnosed kind of cancer globally and the third major cause of cancer death [1, 2]. While significant progress has been achieved in the clinical diagnosis and treatment of HCC, the overall prognosis of HCC patients remains poor. Prognostic assessment is critical for HCC patient monitoring and follow-up, as well as treatment strategy selection [3]. In recent years, the popularization of next-generation high-throughput sequencing technology has greatly enhanced our understanding of cancer biology [4]. The identification of prognostic markers and the development of reliable prognostic prediction models based on the bioinformatic analysis of genomic big data combined with clinicopathological characteristics of patients with HCC offer wide-ranging potential applications [5].

Metabolism is one of the hallmark features of many malignant tumors, including HCC [6]. Peroxisome is a single-layer membrane organelle, which contains many metabolic enzymes and directly participates in various metabolic pathways [7]. A variety of peroxisome enzymes and their activities are altered in HCC and many other types of tumors, and inhibitors of the appropriate enzymes or altered gene expression levels can inhibit or promote tumor growth [8, 9]. Researchers have determined that peroxisomes are implicated in the occurrence and development of a number of cancers. HAO2 is an enzyme that is found in peroxisomes and which catalyzes the oxidation of hydroxy fatty acids to keto acids and hydrogen peroxide [10]. By analyzing transcriptomic data of HCC tissues and their adjacent noncancerous tissues in public databases, a previous study reported significantly lower expression of HAO2 in human HCC tissues, and low expression of HAO2 was related to a worse prognosis in HCC patients [11]. The research on HAO2 is, however, limited. A number of studies have reported a significant reduction in HAO2 expression in drug-induced HCC animal models and human HCC samples, and that overexpression of HAO2 can inhibit the growth of transplanted tumors, indicating that HAO2 may play a tumor-suppressive role in the development of HCC, although its specific mechanism remains unclear [12].

MiR-615-5p is a newly detected endogenous microRNA (miRNA) that has been linked to the development of cancer [13]. A genome-wide DNA methylation sequencing investigation revealed that the degree of methylation in the miR-615-5p promoter region was extremely aberrant, indicating that it may play a role in colon cancer development [14]. Later investigations have shown that miR-615-5p is expressed in cirrhotic and malignant liver tissues in humans, and that overexpression of miR-615-5p may retard the development of HCC [15]. Over the last three years, sequencing study of miRNA groups in a range of diseased and normal tissues has shown that miR-615-5p is inappropriately expressed in carcinogenic tissues and may be utilized as a possible biological marker for the onset and progression of a variety of cancers [16]. However, the function and method by which miR-615-5p contributes to the development of HCC remain unknown.

This study was aimed at determining the prognostic value of HAO2 and examining the expression of HAO2 and its effect on HCC cell proliferation, as well as identifying its potential molecular mechanism of inhibition.

## 2. Materials and Methods

### 2.1. Specimen Collection

23 pairs HCC and adjacent noncancerous liver tissue samples were collected from patients who underwent surgery during Department of Gastroenterology of Henan Provincial People's Hospital from May 2020 to August 2021. After collection, fresh frozen specimens were labeled and immediately placed in liquid nitrogen for freezing and storage and transferred to a -80°C low-temperature refrigerator for long-term storage within 2 hours. All cases were not treated with anticancer therapy before surgery, and the clinicopathological data were complete. The study was conducted with the consent of the Henan Provincial People's Hospital ethics committee, and the patients participated voluntarily with full knowledge of the study content and purpose. The pathological data of the patients were diagnosed by two long-time pathologists under mutually exclusive conditions, and the patients were evaluated for staging based on the TNM grading guidelines prepared by the American Joint Committee on Cancer (AJCC) (AJCC 8th edition, 2018).

### 2.2. Cell Culture and Passaging

The cell lines BEL-7405, Huh7, SNU-387, Hep3B, and THLE-2 were cultured in DMEM complete medium. We set the culture temperature to 37°C and 5% CO_2_. Under normal conditions, passaging treatment was performed when the cell density reached 80-90%. For passaging, the cells were removed from the incubator and washed 3 times with PBS, with uniform and stable rinsing and controlled flow rate. Then, 2 mL of trypsin at a concentration of 0.25% was added and incubated for 3 min, at which point the cells were observed to become round in morphology and digestion was terminated. At this time, the cells were transferred to a 15 mL centrifuge tube, centrifuged at 1000 rpm for 5 min, the supernatant was discarded, and then transferred to a new culture dish according to the ratio of 1 : 2, and the appropriate amount of medium was added and transferred to the cell culture incubator for incubation.

### 2.3. Cell Transfection

Cells were passaged into 6 cm culture dishes and incubated overnight at 37°C. The next day, when the cell density is at 70%-80%, this is the appropriate time for transfection. If the amount of cells is insufficient, wait until the density is appropriate. According to the transfection requirements, prepare the appropriate amount of sterilized centrifuge tubes for preparing the transfection system. Take a 6 cm culture dish as an example, aspirate an appropriate amount of miR-615-5p mimics or inhibitor or HAO2 expression plasmid into a centrifuge tube and add 200 *μ*l Opti-MEM to resuspend slowly. Then, aspirate 2 *μ*l of Lipofectamine 2000 into another centrifuge tube, add 200 *μ*l of Opti-MEM, and slowly resuspend and mix well. Aspirate Lipofectamine 2000 and the target plasmid solution, add to the same centrifuge tube, and let stand for 30 min at room temperature. Take out the cells from the incubator and gently add the mixture dropwise to the culture dish, shaking it slightly a few times. After 4-6 h, aspirate the old medium and continue the culture with new DMEM medium. Cell function or target protein expression can be detected after 48 h. Stable expression cell lines can also be screened using antibiotics.

### 2.4. qRT-PCR

RNA was extracted from tissues and cells by Trizol method. For tissues, 50 g of clinically collected tissue specimens was taken into 1.5 ml enzyme-free EP tubes and sorted and labeled. After adding 1 ml of Trizol and small magnetic beads, the tissues were well ground on a grinder, and the supernatant was transferred to a new EP tube after centrifugation at 4°C for 10 min at 12000 rpm in a low-temperature high-speed centrifuge. For cells, after discarding the medium, wash twice with PBS. After aspirating the PBS from the bottle, 1 ml of Trizol was added, and the cells were lysed sufficiently by pipetting and left for 5 min. High purity total RNA was obtained by chloroform separation, isopropanol precipitation of RNA, and 75% ethanol washing.

260/280 OD values with a ratio in the range of 1.8-2.0 are required. The miR-615-5p expression was detected by Bulge-Loop™ miRNA qRT-PCR kit (licensed patent number: CA201410039162.6). HAO2 expression was detected by the riboSCRIPT qRT-PCR Starter Kit (Guangzhou Ribo Biotechnology Co., Ltd, C11030-1). The primers used in this study are the following: HAO2-F: 5′- GGAGGCAGCTTGATGAGGTT-3′, HAO2-R: 5′- CACCATGTTCACCCTTGCAG-3′; miR-615-5p-F: 5′-ACACTCCAGCTGGGGGGGGTCCCCGGTGCT-3′, miR-615-5p-R: 5′-CTCAACTGGTGTCGTGGAGTCGGCAATTCAGTTGAGGATCCGAG-3′.

### 2.5. Western Blot

Total cellular protein was first extracted. The exponentially growing cells were selected, the stock medium was discarded, and the cells were washed 2-3 times with PBS, i.e., phosphate-balanced saline, with gentle movements. Digestion of the cells was initiated with trypsin and terminated when all cells were rounded and shrunk. Repeated aspiration was performed to free all cells from the wall and centrifuged at 1000 rpm for 10 min in a centrifuge tube. The supernatant was aspirated and 500 *μ*l of RIPA and 5 *μ*l of protease inhibitor were added successively to the centrifuge tube, and the precipitate was lysed after repeated aspiration. After all the lysis was finished, the lysate was transferred to a low-temperature centrifuge at 4°C and centrifuged at 12000 m for 10 minutes, followed by aspiration of the supernatant. Spot electrophoresis was first performed at a constant voltage of 80 V. When the sample was run into the separation gel, electrophoresis was performed at 120 V until the protein bands appeared at the lower edge of the separation gel and then stopped. After electrophoresis is completed, the gel concentrate is removed and the SDS-PAGE gel is transferred to the transfer solution. The PVDF membrane is activated by placing it in methyl alcohol for 1 minute, followed by cleaning. Make a “sandwich structure”, i.e., filter paper+gel+PVDF membrane+filter paper, roll it gently with a glass rod to avoid air bubbles, put it on the sandwich plate in the negative way, and transmold it for 2 hours at a constant current of 250 mA. The transmolded PVDF membranes were treated with TBST containing 5% skim milk powder, and the closure solution was allowed to submerge the membranes for 1 hour at room temperature in a benchtop shaker. The primary antibody is diluted in TBST and the PVDF membranes are incubated with the primary antibody overnight at 4°C. TBST is used to shake and rinse the PVDF membrane for 10 minutes and the rinse should be repeated 3 times. Soak the PVDF membrane with the HRP-containing secondary antibody dilution and incubate at room temperature for 1 h. Shake and rinse the PVDF membrane with TBST for 10 min and repeat the process three times. Chemiluminescent signals were developed using Clarity™ Western ECL Substrate (Bio-Rad, CA, USA).

### 2.6. Cell Proliferation Assay

We measured the proliferative capacity of the cells by CCK-8 assay, clone formation assay, and Edu assay. A very water-soluble orange-yellow methanogenic product is generated by reducing WST-8 by mitochondrial dehydrogenases in the presence of electron-coupled reagents in the CCK-8 experiment. The rate of cell growth is reflected in the color's hue. An enzyme marker is used to determine the OD value at 450 nm, which is used to estimate the number of live cells. Clone formation assay is performed for the formation of clones visible to the naked eye from cultured cells. The rate of clone formation is derived by counting and the proliferation potential of the examined cells is quantified. EdU assay is based on EdU binding to replicating DNA molecules, which is measured by flow cytometry.

### 2.7. Transwell Assay

Matrigel gel was placed in the refrigerator at 4°C one day in advance until it became liquid to be set aside. Matrigel gel was diluted with serum-free medium at a fixation ratio of 8 : 1, and the diluted gel was applied evenly and carefully to the upper chamber surface of the bottom of the invasion chamber. The BEL-7405 and Hep3B cells were cultured with serum-free DMEM medium and starved for 12 h. The cells were then resuspended and a single cell suspension with a cell density of 1 × 10^5^ cells/ml was prepared. Take 200 *μ*l of the above suspension and add it to the upper chamber of the small chamber, avoiding air bubbles when handling. Add 500 *μ*l of medium containing 10 percent extra FBS to the 24-well cell culture plate, then move the upper chamber to the well, and continue the incubation in the incubator. After 48 hours, remove the 24-well plate from the incubator, remove the chambers, place them in a beaker, and wash the chambers repeatedly and gently with phosphate-balanced saline several times. After fixing the cell chamber with 4% paraformaldehyde, 0.1% crystal violet was stained for 20 min and observed by inverted microscopy (X400).

### 2.8. Wound Healing Assay

The cells were detached by trypsinization, resuspended in medium, seeded at a density of 1 × 10^6^ cells/well in a six-well plate, and incubated at 37°C, 5% CO_2_. After the cell monolayer reached confluence, a scratch was made with a sterilized pipette tip, washed with D-Hank's buffer solution and photographed. After culturing for 48 h, the scratch was photographed again. Phase contrast microscope was used to image the scratch photographed twice, and the scratch closure was measured. Each experiment was performed in triplicate.

### 2.9. Dual-Luciferase Reporter System Assay

The binding of miR-615-5p to the target gene was determined by constructing the sequence of the 3′ untranslated region of the target gene HAO2, containing wild type and binding site mutant, into a reporter vector and detecting the change in luciferase activity. Detection was performed by a dual-luciferase reporter gene assay kit.

### 2.10. In Vivo Tumor Formation Assay

The nude mice were kept in an animal room with a constant room temperature of 25°C and a relative humidity of 45-55%, under a 12-h light/12-h dark cycle with ad libitum feeding, and allowed to acclimatize for 1 week prior to the experiment. Briefly, 1 × 10^7^ Hep3B cells stably infected with OV-HAO2 and OV-NC were transplanted into the neck subcutaneous tumors of nude mice and designated as the OV-HAO2 group and OV-NC group, respectively, each group with 4 nude mice. The size of the tumor was measured regularly using a vernier caliper. The volume of the tumor was calculated according to the formula: 1/2 × length × width. After 28 days of breeding, the tumors were dissected and weighed.

### 2.11. Statistical Analyses

Graphpad Prism 8.0 was used to do statistical analysis on the data (GraphPad Software Inc, San Diego, CA, USA). The mean standard deviation was used to represent the relative level of HAO2 expression, the percentage of Edu incorporation, the vitality of cells, the number of cell migrations, the number of cell invasions, the volume of the tumor, and the mass of the tumor. The two independent sample *t*-test was used to compare sample means between groups, and one-way analysis of variance (ANOVA) was used to compare sample means between several groups. A difference of *P* < 0.05 is deemed statistically significant.

## 3. Results

### 3.1. HAO2 Is Underexpressed in HCC

**Figure 1:**
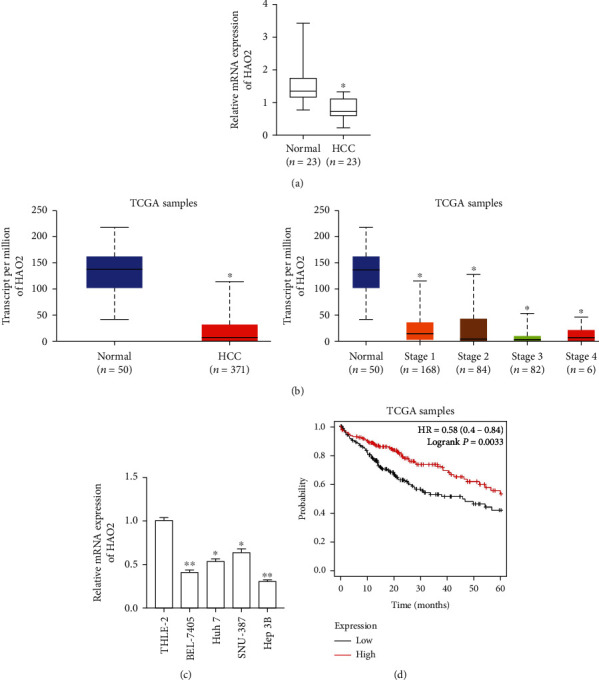
HAO2 is underexpressed in HCC tissues and cell lines. (a) qRT-PCR analysis of mRNA levels of HAO2 in HCC and paracancerous tissues. (b) TCGA database analysis of HAO2 levels in large samples of HCC and paracancerous tissues. (c) qRT-PCR measurement of the HAO2 expression in normal liver epithelial cells THLE-2 and 4 HCC cell lines (Hep3B, BEL-7405, Huh7, and SNU-387). (d) TCGA database analysis of the correlation between the 5-year survival rate and HAO2 expression in HCC patients. ^∗^*P* < 0.05, ^∗∗^*P* < 0.01.

The measurement of the expression levels of HAO2 in 23 HCC tissue samples and matched paracancerous tissue samples by qRT-PCR analysis revealed that HAO2 was lowly expressed in HCC tissues (Figure 1(a)). Also, the results of The Cancer Genome Atlas (TCGA) database [17] analysis of the expression levels of HAO2 in a large sample of HCC and matched paracancerous tissues (Figure 1(b)) showed that HAO2 was strongly underexpressed in HCC tissues, and HAO2 was also noticeably downregulated in tissues of different stages of HCC.

In addition, the results of the qRT-PCR analysis of HAO2 expression levels in normal liver epithelial cells THLE-2 and 4 HCC cell lines (BEL-7405, Huh 7, SNU-387, Hep3B), shown in Figure 1(c), revealed that HAO2 was underexpressed in HCC cell lines to varying degrees. Additionally, the assessment of the relationship between the 5-year survival rate and expression levels of HAO2 in HCC patients through TCGA database revealed that low HAO2-expressing patients had a poorer 5-year survival rate (Figure 1(d)).

### 3.2. HAO2 Overexpression Inhibits HCC Cell Proliferation, Migration, and Invasion

**Figure 2:**
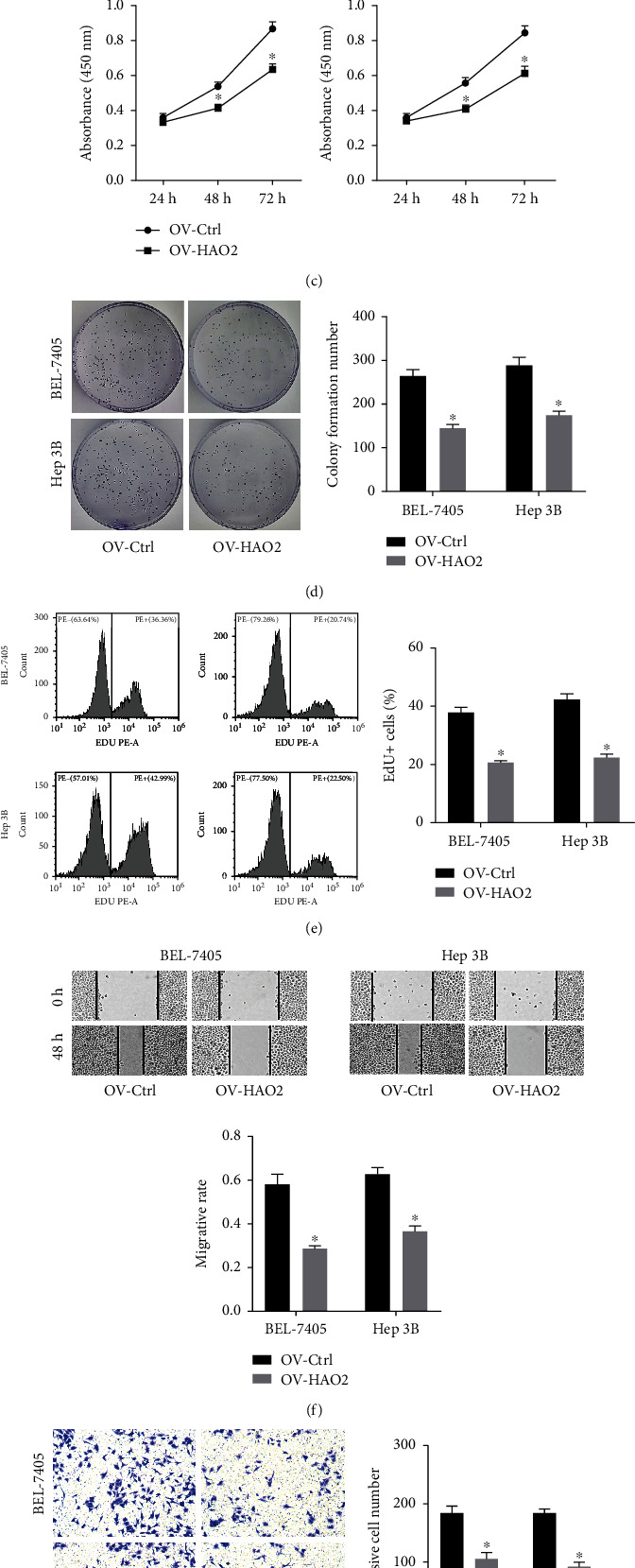
HAO2 overexpression inhibits HCC cell proliferation, migration, and invasion. (a and b) The efficiency of HAO2 overexpression was determined by qRT-PCR and western blot analysis. (c, d, and e) Determination of cell proliferation by the CCK-8 assay, colony formation assay, and Edu incorporation assay. (f and g) Cell migration and invasion ability were measured by wound healing assay and Transwell assays. ^∗^*P* < 0.05, ^∗∗∗^*P* < 0.001.

The biological role of HAO2 was investigated by evaluating the effect of its overexpression in two cell lines with low HAO2 expression, namely, BEL-7405 and Hep3B. The HAO2 overexpression vector used for this purpose was constructed based on pcDNA3.1(+) vector, and the efficiency of HAO2 overexpression was determined by qRT-PCR and western blot. As shown in Figures 2(a) and 2(b), it revealed that the level of HAO2 was increased after the overexpression of HAO2. The results of the CCK-8 assay (Figure 2(c)) showed a marked decrease in the proliferation of HCC cells in the OV-HAO2 group. The results of the colony formation assay (Figure 2(d)) also showed that overexpression of HAO2 inhibited cell proliferation. Additionally, the results of the Edu incorporation assay (Figure 2(e)) also revealed that a significant decrease of the rate of EdU incorporation in the OV-HAO2 group compared with the OV-NC group.

As demonstrated in Figure 2(f), the number of migratory cells was much lower in the elevated-HAO2 cells than in the control cells, suggesting that HAO2 overexpression hindered HCC cell migration. Consistent with the migratory data, the number of invasive cells was also lowered in the HAO2 overexpressing cells (Figure 2(g)). These findings revealed that overexpression of HAO2 impairs cell migration and invasion.

### 3.3. miR-615-5p Targets HAO2 3′UTR Region

**Figure 3:**
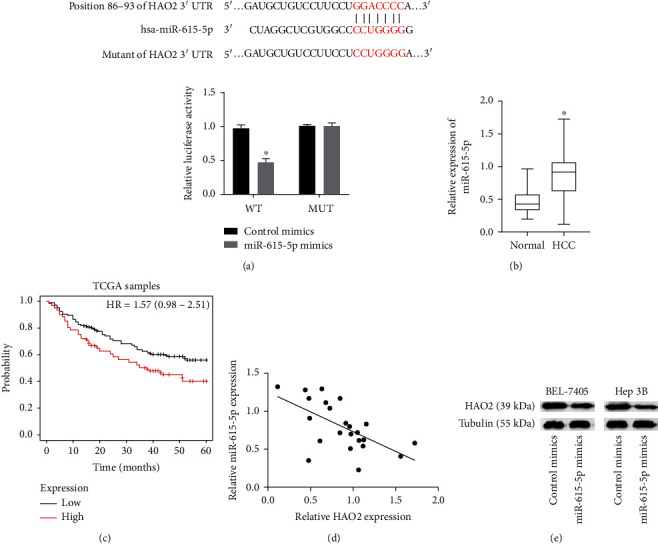
miR-615-5p targets the HAO2 3′UTR. (a) The StarBase online tool and dual-luciferase reporter system experiment indicate that miR-615-5p binds to the HAO2 3′UTR region. (b) The levels of miR-615-5p in 23 pairs of HCC/normal tissues were measured by qRT-PCR. (c) TCGA database analysis of the survival period of high or low miR-615-5p-expressing HCC patients. (d) Negative correlation between the expression of miR-615-5p and HAO2. (e) The expression of HAO2 with miR-615-5p mimics. ^∗^*P* < 0.05.

According to certain research, miR-615-5p was highly expressed in HCC [16]. We predicted that miR-615-5p binds to the HAO2 3′ UTR location using the StarBase online tool and then validated this prediction using the dual-luciferase reporter system assay to demonstrate the impact of miR-615-5p binding to the HAO2 3′ UTR in Hep3B cells (Figure 3(a)). Furthermore, we examined the relationship between miR-615-5p and HAO2 by determining the level of miR-615-5p in 23 pairs of HCC/paracancerous tissues. The qRT-PCR analysis revealed that miR-615-5p expression was high in HCC tissues. (Figure 3(b)). Additionally, an evaluation of the TCGA database revealed that patients with a high level of miR-615-5p expression had a worse survival rate (Figure 3(c)). The analysis of the correlation between the expression level of HAO2 mRNA and miR-615-5p in HCC tissues revealed a negative correlation between expression of miR-615-5p and HAO2, as shown in Figure 3(d). In addition, the results of western blot analysis showed that the level of HAO2 was generally decreased by miR-615-5p mimics (Figure 3(e)).

### 3.4. miR-615-5p Recues the Biological Role of HAO2 in HCC Cells

**Figure 4:**
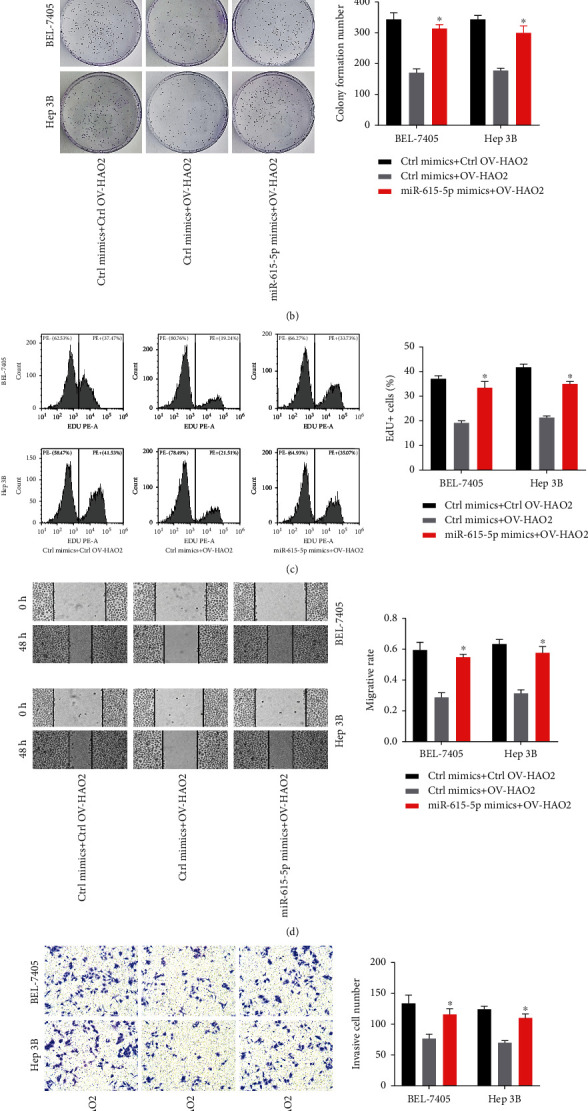
miR-615-5p recues the biological function of HAO2 in HCC cells. (a–c) The proliferation ability was evaluated by CCK-8 cell assay (a), colony formation assay (b), and Edu incorporation assay (c). (d) The migration ability was assessed by wound healing assay. (e) The invasion ability was determined by the Transwell assay. ^∗^*P* < 0.05.

We also checked at whether miR-615-5p affects HAO2's biological function in BEL-7405 and Hep3B cells. The CCK-8 cell assay (Figure 4(a)), colony formation assay (Figure 4(b)), and Edu incorporation test (Figure 4(c)) results demonstrated that overexpression of miR-615-5p partially restored overexpressed HAO2's inhibitory proliferation ability. The wound healing assay results, shown in Figure 4(d), revealed that the number of migrating cells in the miR-615-5p mimics+OV-HAO2 group was considerably higher than in the OV-HAO2 group. Furthermore, the Transwell assay yielded comparable results for the OV-HAO2 and miR-615-5p mimics+OV-HAO2 groups (Figure 4(e)).

### 3.5. Exogenous Expression of HAO2 Weakens the Tumorigenicity of HCC Cells In Vivo in Nude Mice

**Figure 5:**
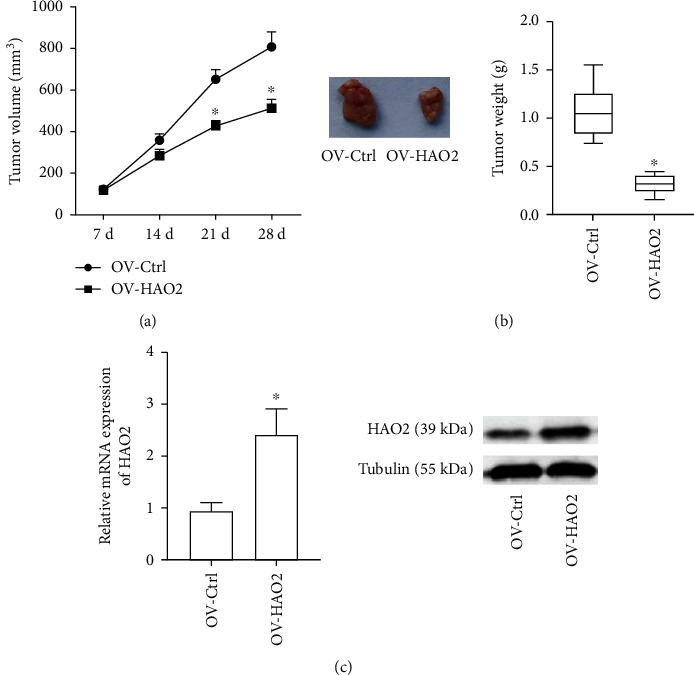
Exogenous expression of HAO2 reduces the tumorigenicity of HCC cells in vivo. (a) The HCC cells in the OV-NC group formed tumors in nude mice, while the tumor growth of the HCC cells in the OV-HAO2 group was significantly inhibited. (b) The tumor weight in the OV-HAO2 group was significantly decreased compared with the OV-NC group. (c) The mRNA and protein levels of HAO2 in tumors of the OV-HAO2 group were overexpressed compared with those of the OV-NC group. ^∗^*P* < 0.05.

Tumor volume was assessed every 7 days after lentivirus-infected cells were injected, and the data was used to create a graph.  Figure 5(a) shows that HCC cells in the OV-NC group produced tumors in mice, but tumor growth in the OV-HAO2 group was considerably suppressed (*P* < 0.05). We excised the tumor tissues and measured the tumor weight 28 days after the injection. The tumor weight of the mice in the OV-HAO2 group was significantly lower than that of the mice in the OV-NC group, as shown in Figure 5(b). The qRT-PCR and Western blot investigations revealed that HAO2 mRNA and protein levels were elevated in tumors from the OV-HAO2 group compared to those from the NC group (Figure 5(c)).

## 4. Discussion

HAO2 is an enzyme that catalyzes hydroxyl-containing fatty acids and directly participates in the oxidative degradation process of fatty acids [18]. In the process of tumor metabolism, fatty acid degradation decreases, and synthesis increases [19–21]. Therefore, the decrease in HAO2 expression in liver cancer conforms to the need for reprogramming tumor cell lipid metabolism [9]. Studies have found decreased expression of HAO2 in liver cancer and renal clear cell carcinoma. Overexpression of HAO2 has also been shown to enhance cellular reactive oxygen species, stimulate lipid oxidation in tumor cells, and limit the formation of liver and kidney tumors [22]; however, the molecular mechanism is unknown.

In this study, we first examined the expression changes of HAO2 in a human HCC tissue genomic database as well as samples of human HCC tissues, and we found that HAO2 levels were lower in patients with HCC tissues, and that low HAO2 expression was associated with a poor prognosis. Inhibition of cell proliferation, migration, and invasion was observed. When HAO2 was overexpressed. When Landgraf et al. [23] sequenced a mammalian genome-wide miRNA collection, they discovered miR-615. Several studies have been conducted in recent years to examine the function and mechanisms mediating the effects of miR-615-5p and discovered that miR-615-5p has a promotion effect on the development of HCC, pancreatic ductal carcinoma, and other cancers [17, 24]. This study compared the expression levels of miR-615-5p in HCC patients' cancer tissues and adjacent tissues and discovered that the expression levels of miR-615-5p in HCC tissues were significantly upregulated, implying that miR-615-5p is associated with the development of HCC, consistent with the results of existing studies [25, 26]. According to the existing literature, AKT2 is the target of miR-615-5p in pancreatic cancer and breast cancer [27, 28]. This study confirmed that miR-615-5p promotes the proliferation of HCC by regulating the level of HAO2, which is a newly identified target of miR-615-5p.

To date, there has been no research report on the molecular mechanism of the inhibitory effect of HAO2 on HCC development or progression. This study conducted an experimental analysis of the inhibitory effect of HAO2 on HCC. It was found that miR-615-5p affects various cell processes by regulating HAO2, which helps to understand the participation of HAO2 and similar peroxidases in possible mechanism of tumor metabolism. The mechanistic part of this study is not in-depth enough. The specific mechanism of miR-615-5p on HAO2 regulation has not been elucidated. Most of the mechanistic experiments were performed in vitro. Our study bears limitations with we cannot obtain a large enough number of clinical case specimens to fully evaluate the clinical abnormal expression of HAO2. The follow-up plan is to improve the in vivo experimental support and perform more in-depth research on the mechanistic part in future studies.

## Data Availability

The data used to support the findings of this study are included within the article.
